# Congenital Afibrinogenemia and Hypofibrinogenemia: Genetic Basis, Pathophysiology, Laboratory Diagnosis, and Management

**DOI:** 10.3390/hematolrep18050072

**Published:** 2026-09-30

**Authors:** Ssakher Alotaibi, Sandrine Meunier, Yesim Dargaud

**Affiliations:** 1 Department of Hematology, Faculty of Medicine, King Abdulaziz University, Jeddah 22252, Saudi Arabia; 2 Reference Center of Hemophilia and Other Rare Bleeding Disorders, Hospices Civils de Lyon, 69002 Lyon, France; sandrine.meunier@chu-lyon.fr (S.M.); gamze.dargaud@chu-lyon.fr (Y.D.)

**Keywords:** afibrinogenemia, hypofibrinogenemia, congenital fibrinogen disorders, fibrinogen, Clauss assay, genotype–phenotype correlation, fibrinogen concentrate, endoplasmic reticulum-associated degradation

## Abstract

Congenital fibrinogen disorders (CFDs) comprise quantitative deficiencies—afibrinogenemia and hypofibrinogenemia—and qualitative defects—dysfibrinogenemia and hypodysfibrinogenemia. This review is confined to the quantitative disorders, and the term quantitative CFDs is used throughout in preference to the wider umbrella term; qualitative defects are considered only insofar as they must be distinguished at the bench. Afibrinogenemia results from biallelic loss-of-function variants in FGA, FGB, or FGG, and is defined by fibrinogen activity below the analytical limit of detection with undetectable antigen; hypofibrinogenemia results from monoallelic variants and is defined by a proportionate reduction in activity and antigen. Several authoritative reviews of this field have appeared recently. The contribution intended here differs in emphasis. Rather than restate the stepwise diagnostic approach, we set out what actually goes wrong at the bench: the analytical variability among fibrinogen methods, the behavior of the Clauss assay near its limit of detection, and overestimation by prothrombin time-derived fibrinogen. We also address interference from anticoagulants and sample factors, the requirement for laboratory-specific reference intervals, the interpretation of discordant results, and the molecular workflow, including copy-number analysis and the handling of variants of uncertain significance. We also address a question that the descriptive literature largely leaves open: why patients carrying identical variants, sometimes within a single family, differ so markedly in phenotype. We propose a four-layer framework—cis-acting effects at the fibrinogen locus, hepatocyte proteostasis and endoplasmic reticulum quality control, trans-acting genetic modifiers, and acquired factors—and review the evidence supporting each. Population genomic data are reconsidered: current estimates derived from gnomAD suggest that predicted-deleterious fibrinogen genotypes are considerably more frequent than clinically ascertained disease, a discrepancy that reflects incomplete penetrance and the limits of in silico prediction rather than a hidden burden of undiagnosed severe disease. Management recommendations are presented with the strength of the supporting evidence made explicit. Fibrinogen concentrate is a first-line replacement. Widely accepted targets are a peak > 1.5 g/L before major surgery, and >1.0 g/L before minor procedures; >1.0 g/L postoperatively until hemostasis, and >0.5 g/L until wound healing; and a trough ≥ 1.0 g/L in pregnancy, rising to ≥1.5 g/L peripartum. All data are derived from expert consensus, registry data, and small interventional series rather than randomized trials, and are presented here as starting points for individualized care. Paradoxical thrombosis, its uncertain mechanism, and the possibility that replacement precipitates it are treated as an unresolved problem rather than a footnote.

## 1. Introduction

Fibrinogen is a 340 kDa hexameric glycoprotein and the principal substrate of thrombin at the terminal stage of coagulation. It is not itself a step in the enzymatic cascade: thrombin, the terminal protease, cleaves fibrinopeptides from it to generate fibrin monomers, which then polymerize into the structural framework of the hemostatic plug; soluble fibrinogen simultaneously serves as the bridging ligand for platelet aggregation. Beyond hemostasis, fibrinogen participates in wound healing, inflammation, angiogenesis and host defense, and these non-hemostatic roles account for several of the clinical features of severe deficiency that bleeding alone does not explain [1,2,3,4].

Inherited defects of fibrinogen have been recognized for a century [5]. They remain among the least common of the rare bleeding disorders, and much of what is known about them derives from case reports, small series, and international registries rather than from controlled studies. This evidential position shapes everything that follows, and we have tried to make it visible rather than to write around it.

### 1.1. Terminology, Definitions, and the Scope of This Review

The umbrella term congenital fibrinogen disorder (CFD) encompasses four entities. Quantitative disorders—afibrinogenemia and hypofibrinogenemia—are characterized by reduced or absent fibrinogen, the residual protein being functionally normal. Qualitative disorders—dysfibrinogenemia and hypodysfibrinogenemia—are characterized by a structurally abnormal molecule, with normal or reduced antigen, respectively [6].

This review addresses the quantitative disorders only. We adopt the term quantitative CFDs throughout and reserve the unqualified abbreviation CFDs for statements that genuinely apply to the whole group. Dysfibrinogenemia and hypodysfibrinogenemia appear only where they must be distinguished from the quantitative disorders during laboratory evaluation [5,6].

The Scientific and Standardization Committee (SSC) of the International Society on Thrombosis and Haemostasis (ISTH) places these four entities in a four-type scheme, in which the quantitative disorders occupy types 1 and 2. Afibrinogenemia (type 1) is defined by fibrinogen activity (Fg:C) below the analytical limit of detection, together with undetectable fibrinogen antigen (Fg:Ag). It is divided into subtype 1A, comprising asymptomatic and bleeding patients, and subtype 1B, afibrinogenemia with phenotypic thrombosis. The two subtypes are not symmetrical: a patient who has sustained a thromboembolic event is assigned to 1B even when hemorrhagic episodes have also occurred, so thrombosis takes precedence in classification. Hypofibrinogenemia (type 2) is defined by Fg:C below the lower limit of the laboratory reference interval, with Fg:Ag reduced in proportion, conventionally expressed as an Fg:C/Fg:Ag ratio ≥ 0.7. It is graded by functional concentration as severe (subtype 2A, <0.5 g/L), moderate (2B, 0.5–0.9 g/L), or mild (2C, 1.0 g/L to the lower reference limit). Subtype 2D is familial hypofibrinogenemia with fibrinogen storage disease (FSD), which requires histological confirmation and is considered in Section 3.3. The qualitative disorders occupy types 3 and 4 and lie outside the scope of this review. Two caveats attach to this scheme. The SSC itself notes that the 0.7 ratio cut-off has never been formally validated, a point returned to in Section 4.5. Hereditary fibrinogen Aα-chain amyloidosis is deliberately excluded from the classification altogether, because routine coagulation assays are unaffected by it; the distinction is made in Section 3.3 [6].

One point of definition deserves emphasis at the outset, because it recurs throughout this review. The severity thresholds above are expressed in absolute concentrations, but the boundary of normality is not. The reference intervals for functional fibrinogen are method-, reagent-, analyzer-, and population-dependent. An adult interval of approximately 1.5–4.0 g/L is widely accepted and is used illustratively here, but every laboratory should verify and apply its own [7].

### 1.2. Epidemiology Reconsidered in Light of Population Genomic Data

Clinically ascertained afibrinogenemia has long been estimated at 1–2 cases per million [8,9]. That figure derives from specialist center caseloads and almost certainly underestimates true genotypic frequency; however, the size and direction of the correction have become clearer only recently.

The Genome Aggregation Database (gnomAD) [10] has been interrogated twice for this purpose. An initial interrogation of approximately 140,000 exomes and genomes suggested that the worldwide prevalence of recessively inherited fibrinogen deficiency might be up to ten-fold higher than the clinical estimate, with significant variations among populations [8]. More recent analysis using gnomAD v4.1.0, which incorporates data from more than 800,000 individuals, estimated that homozygous genotypes predicted to be deleterious could be present on the order of 29 per million, and heterozygous genotypes in as many as 15,000 per million [11].

These figures are genotype-based projections built on in silico deleteriousness prediction, not measurements of disease, and three limitations apply. Computational prediction of pathogenicity has imperfect specificity, so a proportion of variants counted as deleterious could prove benign on functional or segregation evidence [12]. Population databases also overestimate the penetrance of putatively pathogenic variants, because the individuals contributing to them are unselected for phenotype [13]. Most importantly for practice, the great majority of heterozygous carriers have mild hypofibrinogenemia or no phenotype at all and would never come to hematological attention. The wider implications of this discrepancy are taken up in Section 6.

How sensitive these projections are to a single common allele is worth stating concretely, because it bears on how much weight they can carry. In the first gnomAD analysis, the global estimate for recessively inherited fibrinogen deficiency was 10.5 per million; excluding one common FGG variant, p.Ala108Gly, reduced it to 3.2 per million, and in some populations the predicted deficiency became virtually absent [8]. The same variant accounts for the majority of predicted-deleterious alleles among Europeans and is associated with only a modest reduction in fibrinogen concentration. It reappears in Section 2.4 for the same reason: its presence in a patient with hypofibrinogenemia requires assessment rather than automatic attribution. The rise in the more recent estimates is likewise driven in part by the larger and more diverse gnomAD v4 dataset capturing homozygotes not represented in earlier releases [11]. The authors of both analyses stated these cautions themselves; we restate them because the headline figures travel further than the caveats attached to them.

The correct inference is not that a large reservoir of undiagnosed severe disease exists, but that mild hypofibrinogenemia is more common than clinical practice suggests, and that the boundary between a benign biochemical abnormality and a clinically significant deficiency is less distinct than the classification implies.

One frequently repeated figure deserves precise handling rather than dismissal. The statement that fibrinogen gene variants may be present in around 1% of the global population is not unfounded, and it is not a distortion of the literature. It corresponds closely to the carrier frequency reported for the dominantly inherited fibrinogen disorders in the first gnomAD analysis. That analysis put the worldwide figure at approximately 11 per 1000 individuals, ranging from 3 per 1000 in Finns to 1–2 per 100 in non-Finnish Europeans and in Africans and African Americans [8]. The more recent analysis gives a figure of the same order, up to 15,000 per million, or 1.5% [11]. What is misleading is not the number but the use to which it is commonly put. It describes carriage of a single predicted-deleterious allele across all the dominantly inherited fibrinogen disorders taken together—hypofibrinogenemia, dysfibrinogenemia, hypodysfibrinogenemia, fibrinogen storage disease, and hereditary renal amyloidosis. It is neither a prevalence of disease nor a prevalence of the quantitative disorders that are the subject of this review. Quoted without that qualification, a carrier frequency is silently converted into an apparent disease burden, and it is that conversion, rather than the estimate itself, that should not be propagated.

Reported sex distribution has varied widely between series. Most studies find no true sex predilection; the apparent excess of females in some cohorts is best explained by ascertainment, since menorrhagia and adverse obstetric outcomes bring affected women to attention at an age when affected men may remain asymptomatic [14,15]. Because afibrinogenemia is autosomal recessive, its observed frequency is substantially higher in populations where consanguineous marriage is common, a consideration of direct relevance to practice in the Middle East, North Africa, and South Asia [14,16].

### 1.3. Methods of Literature Identification

This is a narrative review, and the search underpinning it is set out here so that its coverage and its limits are transparent. It was not conducted as a systematic review; no protocol was registered, and the reporting conventions for systematic reviews and meta-analyses (PRISMA) are not applicable.

MEDLINE (via PubMed), Embase, Web of Science, and the Cochrane Library were searched from inception to 31 July 2026. The search combined disease terms—afibrinogenemia, hypofibrinogenemia, congenital fibrinogen disorder, congenital fibrinogen deficiency, fibrinogen storage disease—with the gene symbols FGA, FGB, and FGG, and with topic terms covering the domains of this review: Clauss assay, fibrinogen antigen, prothrombin time-derived fibrinogen, reference interval, genotype–phenotype, endoplasmic reticulum-associated degradation, fibrinogen concentrate, cryoprecipitate, prophylaxis, thrombosis, pregnancy, postpartum hemorrhage, and tranexamic acid. The full search strategy for each database is provided in Figure S1 (Supplementary Materials).

Records were restricted to publications in English, with the single exception of a French-language report retained for priority of description [17]. No date limit was applied, because the descriptive literature in this field extends back a century and several foundational molecular papers predate the year 2000. Reference lists of all included reviews, guidelines, and primary studies were hand-searched, and forward citation tracking was performed on the principal guideline documents. Priority was given to guidance from the ISTH SSC, ICSH, UKHCDO, and comparable bodies, to registry and cohort data, and to prospective interventional studies. Case reports were cited only where they document a phenomenon not otherwise recorded, such as juxta-articular bone cysts or hemizygosity at the fibrinogen locus.

Three non-bibliographic sources were interrogated directly: the Human Fibrinogen Database, established by the Groupe d’Étude sur l’Hémostase et la Thrombose and now hosted by the European Association for Haemophilia and Allied Disorders [18], for variant-level data; the Genome Aggregation Database (gnomAD v4.1.0), for population allele frequencies; and the current summaries of product characteristics for the licensed fibrinogen concentrates.

The search was last updated on 31 July 2026, and the manuscript reflects the literature available to that date. Screening and selection were performed by the first author and verified by the co-authors; where the authors disagreed as to whether a source supported a given statement, the statement was either removed or reworded to the weaker claim that the source did support. Selection nonetheless reflects the authors’ judgment rather than a reproducible algorithm, and the consequences of that are stated in Section 6.

## 2. Pathophysiology

### 2.1. Fibrinogen Structure and the Conversion of Fibrinogen to Fibrin

Fibrinogen is a symmetrical dimer of two half-molecules, each composed of one Aα, one Bβ, and one γ chain, encoded by FGA, FGB, and FGG, respectively. The assembled molecule is elongated and trinodular: two distal D regions, each containing the C-terminal globular domains of the Bβ and γ chains, are joined by triple-helical coiled-coil segments to a central E region formed by the disulfide-linked N-termini of all six chains [1,19,20].

**Figure 1:**
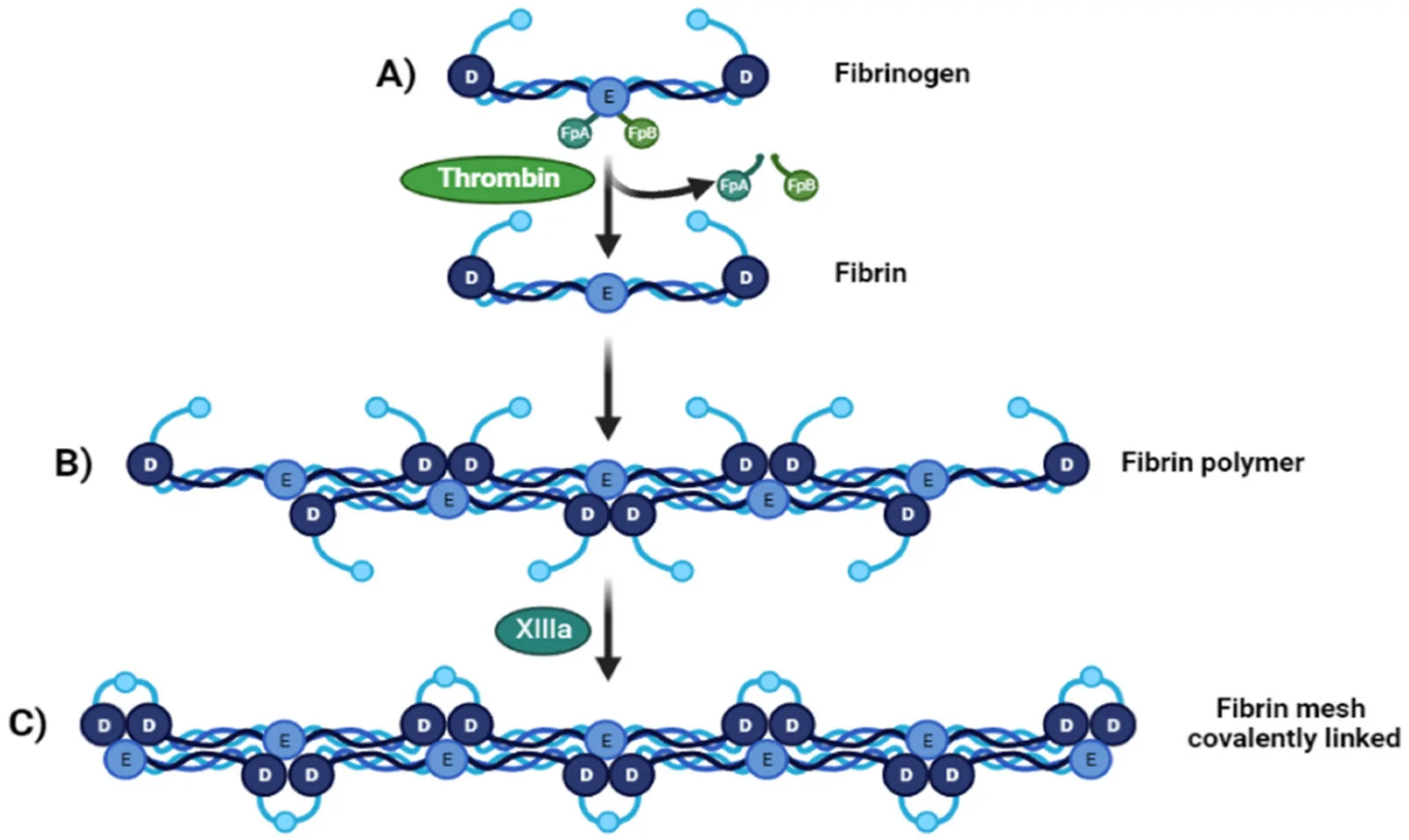
The thrombin-mediated conversion of fibrinogen to fibrin and the cross-linking of the fibrin polymer. (**A**) Thrombin cleaves fibrinopeptides A and B (FpA, FpB) from the central E region, exposing the polymerization sites. (**B**) Fibrin monomers associate in a half-staggered, end-to-middle arrangement to form double-stranded protofibrils that aggregate into the fibers. (**C**) Factor XIIIa introduces covalent cross-links, producing a mechanically and proteolytically stable fibrin mesh. This figure illustrates the conversion of fibrinogen to fibrin only; the biosynthesis and intracellular assembly of fibrinogen are shown separately in Figure 2. (Adapted from Rojas-Murillo et al. [21] under Creative Commons license CC BY 4.0).

Thrombin cleaves fibrinopeptide A from the Aα chains and, more slowly, fibrinopeptide B from the Bβ chains within the central E region. Removal of these peptides exposes polymerization sites—conventionally termed knobs “A” and “B”—that engage complementary holes “a” and “b” in the D regions of adjacent molecules. The resulting half-staggered, double-stranded protofibrils aggregate laterally into fibers, and activated factor XIII introduces covalent γ–γ and α-chain cross-links that confer mechanical and proteolytic stability on the clot [1,19]. This sequence is depicted in Figure 1.

**Figure 2:**
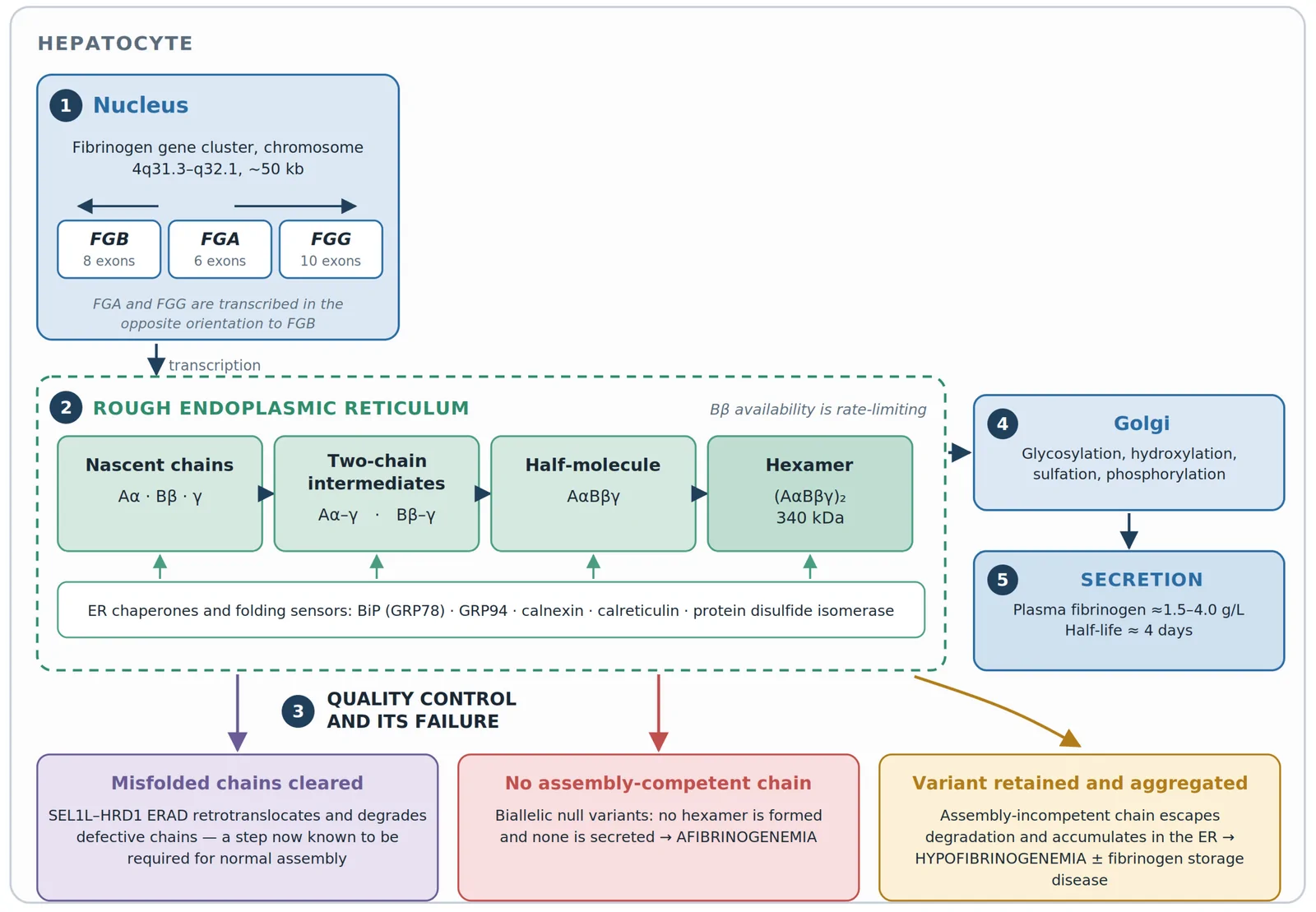
Hepatic fibrinogen biosynthesis, chain assembly, and intracellular quality control, with the three routes to quantitative deficiency. The numbered stages follow the secretory pathway. The fibrinogen gene cluster is transcribed in the hepatocyte nucleus (stage 1); nascent Aα, Bβ, and γ chains are assembled in the endoplasmic reticulum through two-chain intermediates and a half-molecule to the mature hexamer under chaperone supervision (stage 2). Correctly assembled fibrinogen transits the Golgi (stage 4) and is secreted (stage 5). Quality control (stage 3) clears misfolded chains through SEL1L–HRD1 ER-associated degradation, a step now known to be required for normal assembly. Failure at different points produces different phenotypes: the absence of any assembly-competent chain gives afibrinogenemia; the retention and aggregation of an assembly-incompetent variant gives hypofibrinogenemia, and in a subset, fibrinogen storage disease.

One property of fibrin has direct bearing on a later section and is stated precisely here because the distinction is frequently blurred in the literature. It is fibrin, and not fibrinogen, that exerts antithrombin activity. Polymerizing fibrin binds and sequesters thrombin—the property historically termed antithrombin I—and thereby limits further thrombin generation at the site of clot formation [19]. Where fibrin cannot form, this negative feedback is lost. The terminology used in Section 3.4 follows this convention without exception.

### 2.2. Biosynthesis, Chain Assembly, and Intracellular Quality Control

The liver is the only quantitatively significant site of fibrinogen synthesis in humans, a conclusion established definitively by the disappearance of variant fibrinogen from circulation following liver transplantation [22]. The three genes lie within a cluster of approximately 50 kb on the long arm of chromosome 4, ordered FGB–FGA–FGG from the centromere to the telomere at 4q31.3–q32.1, with FGA and FGG transcribed in the opposite orientation to FGB. FGA contains 6 exons, FGB 8, and FGG 10 [23,24]. Coordinated expression is achieved through shared regulatory elements and the local chromatin architecture, and hepatic transcription is upregulated by interleukin-6 during the acute-phase response [25].

Assembly proceeds in the endoplasmic reticulum (ER) through an ordered pathway (Figure 2). Nascent chains are translocated co-translationally and form two-chain intermediates—Aα–γ and Bβ–γ—which acquire the third chain to give the half-molecule AαBβγ; two half-molecules then dimerize to the mature hexamer, stabilized by 29 disulfide bonds. Assembly is assisted by ER chaperones, including BiP (GRP78), GRP94, calnexin, calreticulin, and protein disulfide isomerase. The availability of the Bβ chain is rate-limiting for hexamer formation, which explains why FGB variants that impair chain folding produce disproportionate reductions in secreted protein [26,27]. The assembled hexamer transits the Golgi, where it undergoes glycosylation, hydroxylation, sulfation, and phosphorylation, and is then secreted.

The quality-control arm of this pathway has been substantially clarified. Variant chains that cannot be incorporated into the hexamer are recognized and degraded rather than secreted, and it is this disposal step—not a failure of synthesis—that accounts for the absence of circulating protein in many cases [26]. More recently, the SEL1L–HRD1 complex of ER-associated degradation (ERAD) has been shown to degrade misfolded endogenous Aα, Bβ, and γ chains. Critically, it has also been shown to be indispensable for the formation of a functional hexamer: loss of this complex in hepatocytes produces fibrinogen-positive inclusion bodies, and the same machinery attenuates the pathogenicity of disease-causing γ-chain variants [28]. Fibrinogen biogenesis, therefore, depends not only on the integrity of the three chains but on the capacity of the hepatocyte to dispose of the defective ones.

Two clinically important consequences follow. First, whether a given variant behaves recessively or exerts a dominant-negative effect depends on whether the abnormal chain is efficiently degraded or instead sequesters normal partner chains within the ER. Second, when an assembly-incompetent chain escapes degradation and aggregates, the result is intracellular accumulation—the mechanism of fibrinogen storage disease (Section 3.3). Both observations bear directly on phenotypic variability.

### 2.3. Non-Hemostatic Functions of Fibrinogen

Circulating fibrinogen is present at approximately 1.5–4.0 g/L, with a plasma half-life of about four days, and rises several-fold during inflammation, infection, tissue injury, and pregnancy [2,22]. Soluble fibrinogen bridges activated platelets through the integrin αIIbβ3 (glycoprotein IIb/IIIa) receptor, and fibrin anchors platelets to the injured surface in concert with von Willebrand factor [19]. The γ-chain C-terminus provides binding sites for integrin receptors, growth factors, and coagulation factors, and mediates several of these interactions [29].

Fibrin(ogen) also serves as a provisional matrix in wound repair, as a scaffold and ligand in leukocyte recruitment and innate immunity, and as a binding partner for fibroblast growth factor-2, the vascular endothelial growth factor, and interleukin-1β, thereby modulating endothelial proliferation and inflammation [3,4]. These functions are not incidental to the clinical picture. Impaired wound healing, the bone cysts described in Section 3.1, and the placental attachment failure underlying early pregnancy loss in afibrinogenemia are all consequences of the absence of fibrin as a structural and signaling substrate, rather than of bleeding, as such.

### 2.4. The Molecular Genetics of the Quantitative Fibrinogen Disorders

More than 200 causative variants have been reported across the three fibrinogen genes, cataloged in the Human Fibrinogen Database and in general variant repositories [18]. The mutational architecture of the quantitative disorders is now reasonably well defined and differs materially from the account given in the older literature.

In the largest systematic analysis of mutational epidemiology, afibrinogenemia was almost invariably caused by null alleles—frameshift, nonsense, or splice-site variants predicted to abolish production of the corresponding chain—accounting for 98.6% of cases, with approximately 85% located in FGA. Hypofibrinogenemia, by contrast, was predominantly caused by missense variants in FGB or FGG, which together accounted for some 54% of cases [30]. This pattern is consistent across subsequent series and registry data [23,31].

Two recurrent FGA alleles account for a disproportionate share of afibrinogenemia and should be screened first in a new kindred: a deletion of approximately 11 kb encompassing most of the gene [32], and the splice-donor variant c.510+1G>T (historically IVS4+1G>T), which activates multiple cryptic donor sites [33,34].

A claim that recurs in the earlier literature requires correction. It is not the case that all pathogenic point variants identified in FGA cause C-terminal truncation. The accurate statement is threefold. The great majority of FGA variants causing afibrinogenemia are null alleles, and FGA is by a wide margin the commonest afibrinogenemia locus. Missense variants in FGA do occur but are chiefly associated with other phenotypes: with dysfibrinogenemia when they affect the fibrinopeptide A cleavage region, and with hereditary renal amyloidosis when they affect the Aα-chain C-terminus [35]. Finally, truncating variants of the αC region are not uniformly severe, because the C-terminal αC domain is dispensable for hexamer assembly and secretion even though its loss alters clot architecture. Current variant databases should be consulted directly rather than relying on summary statements of this kind, which date quickly.

FGB variants are less common but mechanistically informative. Missense changes clustering in the C-terminal globular βC domain impair folding and prevent secretion, and because the Bβ chain is rate-limiting, they can produce severe deficiency; several such variants have been shown by expression studies to be retained intracellularly [27]. FGG variants are predominantly missense, are concentrated in the γ-chain D region, and are the commonest cause of hypofibrinogenemia; among them, γ375Arg→Trp (fibrinogen Aguadilla) is the archetypal cause of fibrinogen storage disease [36].

Three points of practice arise. First, variant nomenclature in this field is treacherous: the older literature uses mature-protein numbering while HGVS convention uses precursor numbering, so that the same FGG change appears as γ375Arg→Trp and as p.Arg401Trp. Reports should specify the reference transcript and the numbering convention used. Second, common variants complicate interpretation; FGG p.Ala108Gly, for instance, is frequent enough in the general population that its presence in a patient with hypofibrinogenemia requires careful assessment rather than automatic attribution [37]. Third, a single heterozygous variant identified alongside an unexpectedly severe phenotype should prompt a search for a second event—a deletion on the other allele, or hemizygosity arising from a larger chromosomal deletion—rather than acceptance of an incomplete explanation [37].

### 2.5. Genotype–Phenotype Relationships: A Framework for Phenotypic Variability

**Figure 3:**
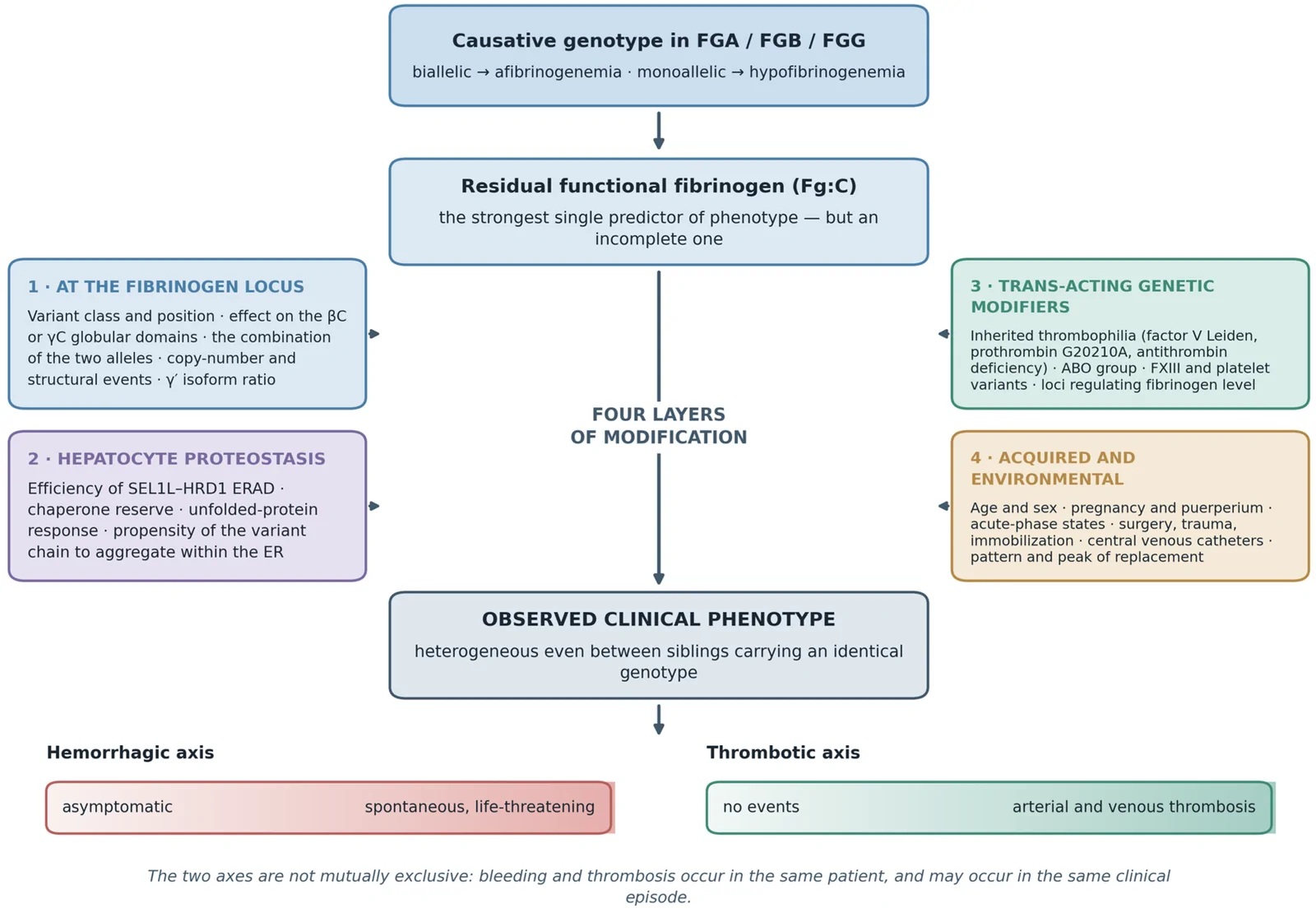
A four-layer framework for phenotypic variability in the quantitative fibrinogen disorders. The residual functional fibrinogen is the strongest single determinant of phenotype but is an incomplete predictor at the level of the individual patient. Four layers of modification—cis-acting effects at the fibrinogen locus, hepatocyte proteostasis, trans-acting genetic modifiers, and acquired factors—act between the genotype and observed phenotype, which is distributed across the two non-exclusive axes of hemorrhagic and thrombotic risk. The figure presents a conceptual framework for organizing current knowledge and for generating testable hypotheses; it is not a validated predictive model and has not been tested against any cohort.

The most striking clinical feature of these disorders is not their severity but their inconsistency. Patients carrying the same variant, including siblings within a single kindred, may differ substantially in bleeding frequency, in thrombotic events, and in obstetric outcome [38,39]. The literature acknowledges this but rarely pursues it. We propose that the variability is better understood as the product of four superimposed layers of modification, summarized in Figure 3.

We state at the outset what this framework is and what it is not. It is a conceptual scheme, offered to organize existing observations, to make explicit the level at which each proposed modifier is thought to act, and to generate testable hypotheses. It is not a validated predictive model. It has not been fitted to or tested against any cohort; it assigns no relative weight to the four layers, and the layers are not mutually exclusive, since a single patient may be modified at several levels at once. It should not be used to counsel an individual patient about prognosis, and the evidence supporting each layer differs considerably in strength, as the paragraphs below make clear.

Layer 1: cis-acting effects at the fibrinogen locus. Variant class and position matter beyond the simple null-versus-missense dichotomy. Variants affecting the βC or γC globular domains impair assembly and secretion, whereas αC truncations permit secretion of a structurally altered molecule. The combination of the two alleles, rather than either alone, determines the residual output; compound heterozygotes for a null and a hypomorphic allele may have measurable fibrinogen, whereas a null homozygote has none. Structural and copy-number events at the locus, and variation in the ratio of the γ′ splice isoform, add further heterogeneity [23,37].

Layer 2: hepatocyte proteostasis and ER quality control. This layer is the least discussed and, on current evidence, among the most likely to be important. Whether a variant chain is degraded, secreted, or retained depends on the efficiency of ER quality control in that individual, and this efficiency is itself variable [26,28]. The clearest clinical illustration is fibrinogen storage disease: among carriers of the same FGG variant, some develop hepatic inclusions and progressive liver disease, while others have isolated hypofibrinogenemia with normal liver histology. It has been proposed that those who develop liver disease are predisposed by less efficient ER quality-control or aggregate-clearance mechanisms, potentially through variation in a second gene [40]. The demonstration that SEL1L–HRD1 ERAD both prevents fibrinogen aggregation and attenuates the pathogenicity of γ-chain mutants provides a concrete molecular candidate for this predisposition [28].

Layer 3: trans-acting genetic modifiers. Coexisting inherited thrombophilia may transform the phenotype. The coexistence of afibrinogenemia with antithrombin deficiency, for example, has been associated with a course dominated by recurrent arterial and venous thrombosis alongside life-threatening bleeding, a combination that makes any single management strategy inadequate. Factor V Leiden, prothrombin G20210A, ABO blood group, factor XIII, platelet-function variants, and the large number of loci that regulate plasma fibrinogen concentration in the general population all plausibly contribute [11,41]. Systematic evaluation of these modifiers in fibrinogen-deficient cohorts has not been performed, and this is a tractable research question.

Layer 4: acquired and environmental factors. Age, sex, pregnancy and the puerperium, acute-phase states, surgery, trauma, immobilization, indwelling central venous catheters, and the pattern and peak level of fibrinogen replacement all modulate the phenotype. Several of these are iatrogenic and are, therefore, the layer most amenable to modification [42].

Two conclusions follow, and they are less comfortable than the descriptive literature implies. Residual fibrinogen activity is the strongest available predictor of bleeding at the group level, but its performance in the individual is modest: the analysis of the Prospective Rare Bleeding Disorders Database found that bleeding severity grade agreed with the factor activity threshold in only about half of patients with quantitative defects [31,43]. Moreover, no validated model exists that predicts, from genotype and baseline fibrinogen, which patient will bleed catastrophically, which will thrombose, and which will remain well. Statements of the genotype–phenotype correlation in this field remain descriptive rather than predictive, and should be presented to patients as such.

## 3. Clinical Presentation

The clinical spectrum extends from complete absence of symptoms to spontaneous, life-threatening hemorrhage, and—paradoxically—to arterial and venous thrombosis, sometimes in the same patient. Acquired causes of hypofibrinogenemia must be excluded before a congenital disorder is diagnosed; these include decompensated liver disease, disseminated intravascular coagulation and consumption, hemodilution and massive transfusion, thrombolytic therapy, and L-asparaginase. Conversely, pregnancy, inflammation, malignancy, smoking, and the acute-phase response raise fibrinogen and may mask mild deficiency [6,42].

### 3.1. Congenital Afibrinogenemia

Afibrinogenemia is the severe end of the spectrum. Bleeding typically declares itself in the neonatal period, classically as prolonged bleeding from the umbilical stump, which occurs in a substantial minority and should always prompt coagulation testing. The subsequent pattern comprises mucocutaneous bleeding, epistaxis, musculoskeletal bleeding, including hemarthrosis, menorrhagia, gastrointestinal, and genitourinary bleeding, disproportionate bleeding after minor trauma or surgery, and—uncommonly but with disproportionate consequence—intracranial hemorrhage [9,16].

The international QualyAfib study of 204 patients from 25 countries provides the most complete contemporary picture and is worth quoting precisely because it corrects an impression of relative benignity that pervades older accounts. One-third of patients experienced at least one bleed per month; 23% had a history of cerebral bleeding; 18.1% had experienced a venous or arterial thrombotic event; and approximately 35% were receiving prophylaxis. The severity of the clinical phenotype was associated with impaired health-related quality of life, particularly in younger patients [14].

Musculoskeletal bleeding is common and, in some series, exceeds mucosal bleeding in frequency [9]. Menorrhagia is frequent and may be the only manifestation in affected women [15]. Asymptomatic intervals between bleeds are characteristic and should not be interpreted as a mild phenotype. Wound healing is impaired independently of bleeding, reflecting the role of fibrin as a provisional matrix [3].

Two less familiar complications deserve mention. Spontaneous splenic rupture is reported more often in afibrinogenemia than in other bleeding disorders and carries high mortality; the proposed sequence is minor trauma or infection causing splenomegaly, followed by microvascular injury, subcapsular hematoma, and capsular rupture in the absence of effective hemostasis [16]. Skeletal involvement, first described in 1967 [17], comprises juxta-articular bone cysts arising from intraosseous hemorrhage with subsequent trabecular remodeling; most cases develop during childhood, suggesting a relationship with skeletal growth, and cases continue to be reported [44,45].

### 3.2. Congenital Hypofibrinogenemia

Hypofibrinogenemia produces a graded phenotype that broadly tracks residual fibrinogen activity. Patients with mild hypofibrinogenemia are frequently asymptomatic and are identified incidentally on preoperative screening or family study. Bleeding, when it occurs, is usually provoked rather than spontaneous, localized to the site of trauma or surgery, and comprises menorrhagia, mucosal bleeding, and, less often, musculoskeletal or gastrointestinal bleeding. Severe hypofibrinogenemia may be clinically indistinguishable from afibrinogenemia. Obstetric complications—placental abruption, postpartum hemorrhage, and pregnancy loss—occur across the range and are not confined to the severe end [9,31,46].

A point of nomenclature should be stated precisely. Although afibrinogenemia and hypofibrinogenemia have historically been considered distinct clinical entities, current evidence indicates that they represent parts of a phenotypic spectrum associated with variants in the same fibrinogen genes. This formulation is deliberately weaker than the assertion that they represent “the phenotypic spectrum of the same genotypes”. The genetic overlap is real, but the two disorders differ systematically in inheritance pattern, in bleeding risk and in management requirements, with afibrinogenemia presenting as the more severe, spontaneously bleeding phenotype [31,47].

### 3.3. Fibrinogen Storage Disease, and Its Distinction from Hereditary Renal Amyloidosis

A subset of patients with hypofibrinogenemia—almost exclusively those carrying particular FGG missense variants—develop fibrinogen storage disease, in which the variant chain is retained within the hepatocyte ER and accumulates as characteristic inclusion bodies. The clinical spectrum ranges from asymptomatic transaminase elevation to fibrosis and cirrhosis. Diagnosis rests on a liver biopsy showing eosinophilic intracytoplasmic inclusions that are periodic acid–Schiff-negative after diastase treatment and immunoreactive for fibrinogen but not for α1-antitrypsin, together with molecular confirmation [36,40]. The variability in hepatic involvement among carriers of the same variant is the clinical signature of the proteostatic modifier layer discussed in Section 2.5.

A distinction that has been elided in some accounts should be made explicitly. Hereditary renal amyloidosis caused by FGA variants affecting the Aα-chain C-terminus is a separate disease, characterized by glomerular deposition of amyloid fibrils derived from the variant Aα chain and presenting with proteinuria, hypertension, and progressive renal failure. It is not a consequence of hypofibrinogenemia, and patients are typically not fibrinogen-deficient [35]. Conflating the two misrepresents both.

### 3.4. Thrombosis: Frequency, Mechanism, and What Remains Unexplained

That patients with no measurable fibrinogen suffer arterial and venous thrombosis remains one of the more counterintuitive observations in hemostasis. It is not rare: 18.1% of patients in the QualyAfib cohort reported a thrombotic event [14], and thrombosis is a recognized cause of morbidity across registry series [16,31].

Several mechanisms are proposed, and the terminology used here follows Section 2.1 precisely. Fibrin—not fibrinogen—binds and sequesters thrombin and limits prothrombin activation; in the absence of fibrin formation, this negative feedback is lost, leaving unopposed thrombin available to activate platelets, the endothelium, and vascular smooth muscle, and to drive intimal hyperplasia through platelet-derived growth factor release [16,19]. Platelet aggregation is not abolished in afibrinogenemia, because the von Willebrand factor can substitute for fibrinogen as the αIIbβ3 ligand, so a platelet-rich thrombus can form without fibrinogen. Superimposed on these intrinsic mechanisms are iatrogenic contributions: fibrinogen replacement transiently generates a substrate-replete state that may be prothrombotic, particularly at supraphysiological peaks, and indwelling central venous catheters used to deliver it are themselves a thrombotic risk [14,42].

We state plainly what is not known. Whether replacement therapy causes thrombosis in these patients, or whether thrombosis occurs in a subgroup who would have thrombosed regardless, has not been resolved: the association is derived from observational data in which the sickest patients receive the most product. No validated tool stratifies thrombotic risk in this population, and the presence or absence of a thrombotic phenotype cannot currently be predicted from genotype or fibrinogen level. The ISTH subtype 1A/1B distinction is descriptive and retrospective, assigning a patient to 1B only once a thrombotic event has occurred; it does not identify at-risk patients prospectively. This is arguably the single most important unmet need in the field, and it is addressed further in Section 6 and Section 7.

### 3.5. Obstetric Manifestations

Fibrinogen is required for placental attachment and for the maintenance of maternal–fetal vascular integrity, and pregnancy in quantitative CFDs is correspondingly high-risk. A systematic review of the published literature reported a miscarriage rate of approximately 43%, most losses occurring in the first trimester, with increased rates of placental abruption and postpartum hemorrhage [46]. Additional recognized complications include first-trimester bleeding, retrochorionic hematoma, fetal growth restriction, preterm delivery, and postpartum thrombosis [15,48].

These figures should be read with their limitations in view. The obstetric literature in this field consists overwhelmingly of case reports and small series, and is subject to marked publication bias: uncomplicated pregnancies in women with mild hypofibrinogenemia are unlikely to be reported. The 43% figure is, therefore, best interpreted as characterizing the severe end of the spectrum, particularly untreated afibrinogenemia, rather than as an estimate applicable to all women with a quantitative fibrinogen disorder. Management is addressed in Section 5.5.

### 3.6. Comparative Summary

Table 1 summarizes the clinical, laboratory, and management differences between the two disorders. It is presented because the two are frequently discussed together in a way that obscures differences of practical importance.

## 4. Diagnosis

**Figure 4:**
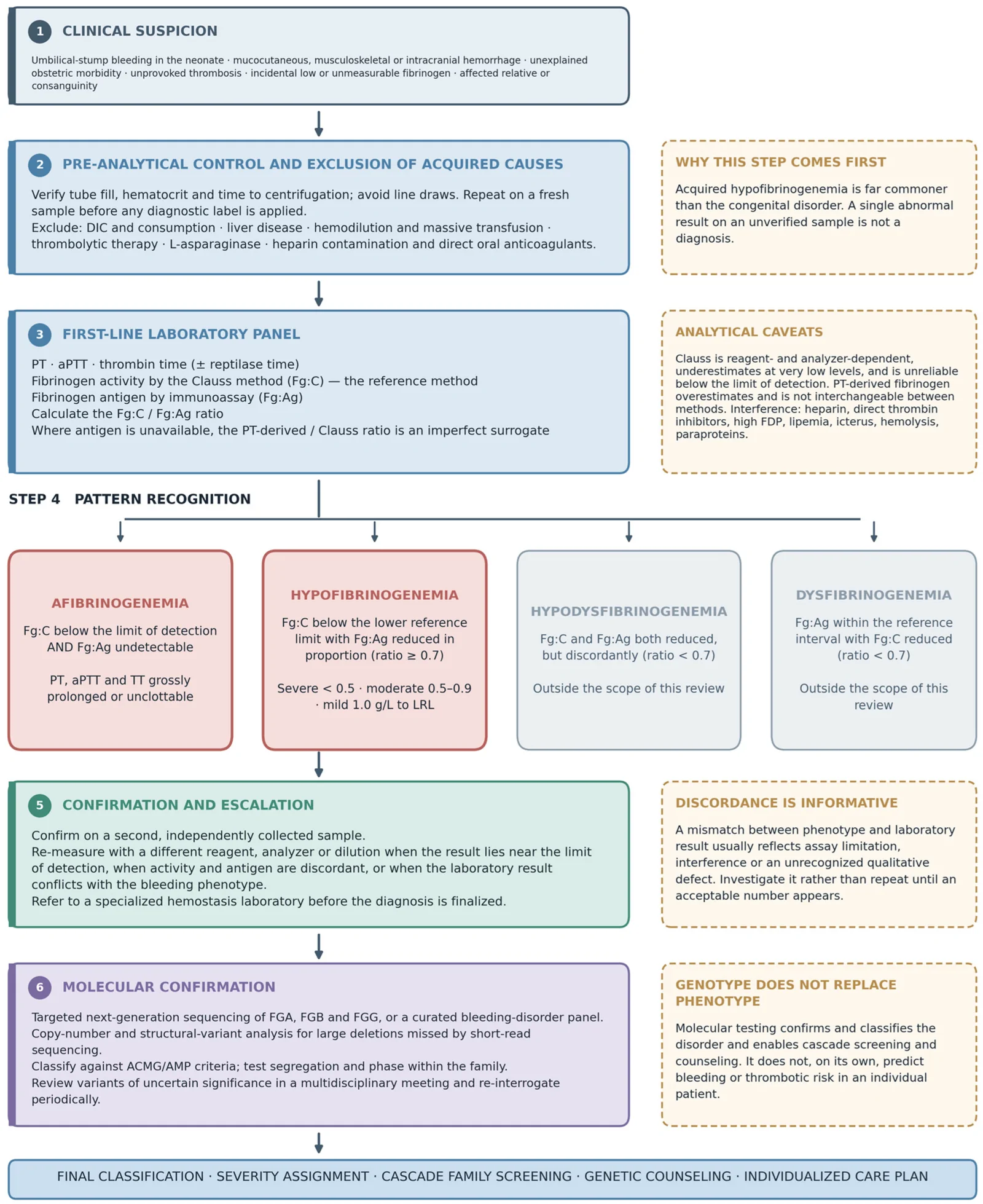
The proposed stepwise diagnostic algorithm for suspected congenital fibrinogen disorders, incorporating analytical pitfalls and escalation pathways. The steps proceed from clinical suspicion through pre-analytical control, first-line testing, pattern recognition, confirmation, and molecular diagnosis. The two entities within the scope of this review are outlined in bold; the qualitative disorders are shown for differential diagnosis only. The annotations to the right of each step identify the errors that most commonly occur at that point. Fg:C, fibrinogen activity; Fg:Ag, fibrinogen antigen; LRL, lower reference limit; and FDP, fibrin degradation products.

Diagnosis of a quantitative fibrinogen disorder is conceptually simple and practically difficult. The conceptual scheme—measuring activity and antigens, computing their ratio, and confirming genetically—is well established and is reproduced accurately in existing reviews [6,49]. What is less often set out is how the measurements behave when they are made at the extremes of the analytical range, in the presence of interfering substances, or on samples that were imperfectly collected. This section is organized around those problems, because they are what generate diagnostic error in practice. The overall approach is summarized in Figure 4.

### 4.1. When to Suspect the Diagnosis

Several presentations warrant investigation. In the neonate, bleeding from the umbilical stump is the classic trigger. At any age, investigation is indicated for mucocutaneous, musculoskeletal, or intracranial bleeding without an obvious cause, for bleeding disproportionate to trauma or surgery, and for menorrhagia with a suggestive family history. Obstetric triggers are recurrent first-trimester pregnancy loss, placental abruption, and postpartum hemorrhage. Investigation is also warranted for unprovoked thrombosis in a young patient, particularly where there is also a bleeding history, and for the incidental finding of a low or unmeasurable fibrinogen or of an unexplained prolongation of PT, aPTT, and thrombin time. A structured bleeding assessment tool improves the objectivity of the clinical assessment and has been evaluated specifically in fibrinogen deficiency, although its discriminating power in mild cases is limited [50]. Consanguinity or an affected relative substantially raises the pre-test probability.

### 4.2. Pre-Analytical Variables

No interpretation is safe on a poorly collected sample, and pre-analytical error is the commonest reason for a spurious fibrinogen result. Citrate tubes must be filled to the mark, since underfilling raises the effective citrate concentration and prolongs clotting times; the citrate-to-plasma ratio should be adjusted when the hematocrit exceeds approximately 0.55. Samples drawn through heparinized lines are a recurrent source of falsely low Clauss results. Centrifugation conditions, time to testing, and freeze–thaw history all influence the result, and standardized recommendations exist [51].

The practical rule that follows from this is stated in Figure 4 and repeated here because it is frequently disregarded: a single abnormal fibrinogen result on an unverified sample is not a diagnosis. Confirmation on a second, independently collected specimen should precede any diagnostic label, and certainly any decision to proceed to genetic testing.

### 4.3. The Clauss Assay: The Principle, Performance, and Limitations

The Clauss method remains the reference technique for functional fibrinogen. Diluted plasma is clotted with a high concentration of thrombin, so that the clotting time depends on the fibrinogen concentration and is largely independent of the thrombin concentration and of most inhibitors; the result is read from a calibration curve constructed from dilutions of a reference plasma [7].

Its limitations are real and are underemphasized in the clinical literature. Reagent thrombin concentrations differ substantially between manufacturers, calibrators are not traceable to a single international standard, and endpoint detection may be mechanical or optical. The consequence is appreciable between-laboratory and between-method variation, such that results from different centers are not directly interchangeable and serial monitoring is best performed on a single platform [7,52].

Two performance characteristics matter specifically in this patient group. First, the assay loses accuracy at very low concentrations, and in patients with severe hypofibrinogenemia, it tends to underestimate. Repeating the measurement at a lower plasma dilution extends the usable range and should be requested when the initial result falls near the limit of detection. Second, the distinction between “below the limit of detection” and “absent” is analytical, not biological. A result reported as undetectable establishes only that the concentration lies below what that method can measure; distinguishing true afibrinogenemia from severe hypofibrinogenemia requires a sensitive antigen assay, and ultimately genotype [6,7].

Optical endpoint detection is additionally vulnerable to sample factors—lipemia, icterus, and hemolysis—which mechanical detection tolerates better. Where these are present, and the result is implausible, repetition on a mechanical analyzer is appropriate.

### 4.4. Prothrombin Time-Derived Fibrinogen

The prothrombin time-derived fibrinogen (PT-Fg) is computed from the magnitude of the optical or mechanical signal change during the PT reaction, calibrated against plasmas of known fibrinogen concentration. It is attractive because it is generated without additional reagent cost or sample volume, and it is widely reported by default.

It should not, however, be used to diagnose or exclude a fibrinogen disorder. PT-Fg systematically overestimates fibrinogen relative to the Clauss method; the discrepancy is greatest at low concentrations and in the presence of a structurally abnormal molecule, and results from different PT-Fg methods are not interchangeable [7,52,53]. In dysfibrinogenemia, the difference can be striking, with PT-Fg values within the reference interval when the Clauss value is below 0.5 g/L [52]. Current ICSH guidance is explicit that PT-Fg results should be interpreted with caution and are not equivalent to Clauss values [7].

Where an antigen assay is unavailable, the ratio of PT-Fg to Clauss fibrinogen can be used as a crude screen for a qualitative defect, with a ratio above approximately 1.43 suggesting discordance that warrants further evaluation. This is a surrogate of convenience rather than a validated diagnostic criterion, and referral for antigen measurement remains preferable.

### 4.5. The Fibrinogen Antigen and the Activity-to-Antigen Ratio

The immunological measurement of fibrinogen antigen—by immunoturbidimetry, nephelometry, radial immunodiffusion or ELISA—is what separates quantitative from qualitative deficiency, and it is indispensable to the classification. An Fg:C/Fg:Ag ratio ≥ 0.7 indicates a quantitative defect; a ratio < 0.7 indicates a qualitative or combined defect. Some laboratories apply a lower cut-off of 0.55, and the choice of threshold alters the classification of borderline cases [6,7].

Antigen assays have limitations of their own that are seldom stated, and these are documented in the analytical literature rather than being matters of opinion. Immunological methods are not standardized across platforms. The British Committee for Standards in Haematology guideline on fibrinogen assays sets out the reasons. Immunological methods measure total immunoreactive fibrinogen irrespective of clottability, and therefore also detect circulating fibrinogen fragments and degradation products. Their results additionally depend on the specificity of the antiserum employed, so that values generated by different immunoassays are not interchangeable [54]. Calibration is subject to the same problem. A formal performance evaluation of commercial fibrinogen reference preparations found appreciable differences among them, and among them and the International Standard for fibrinogen in plasma, so that a value assigned against one calibrator is not directly transferable to another [55,56].

Two consequences follow specifically for the activity-to-antigen ratio. First, whether an antigen assay detects a truncated or structurally aberrant molecule depends on whether the epitopes recognized by the antiserum are retained in that molecule. A variant that has lost the relevant epitopes will be under-measured. This depresses the antigen value and inflates the calculated ratio and can misclassify a qualitative defect as a quantitative one. Second, the ratio is a quotient of two independently calibrated measurements and therefore carries the imprecision of both. That imprecision is not trivial. The largest external quality assessment of fibrinogen methods distributed two identical vials of normal plasma to more than 3500 laboratories and evaluated 50 distinct methods. The all-method bias was 8.3%, with method-specific biases spanning 0% to 27%; the all-method coefficient of variation was 7.7%, with method-specific values spanning 0.7% to 25.8%. Photo-optical endpoint detection performed appreciably worse than mechanical detection [57]. Those figures were obtained on normal plasma, and performance at the low concentrations that characterize these patients is worse still.

Three practical rules follow. Activity and the antigen should be measured in the same laboratory, on the same specimen, using methods whose calibration is documented. Ratios falling close to the 0.7 decision threshold should be repeated and interpreted conservatively rather than acted upon. And a ratio should never be computed from an activity result and an antigen result taken from different reports or different centers, because the resulting figure has no definable analytical uncertainty.

### 4.6. Analytical Interferences, Including Anticoagulants

Interference is the most frequent cause of a fibrinogen result that does not fit the patient, and Table 2 sets out the principal sources with their effects and mitigations.

Anticoagulants merit specific comment because they are so often present. Unfractionated heparin at high concentration and direct thrombin inhibitors, including dabigatran, argatroban, and bivalirudin, can produce falsely low Clauss fibrinogen values, the magnitude depending on the reagent’s thrombin concentration and on whether a heparin neutralizer is incorporated [7,58]. Direct factor Xa inhibitors have comparatively little effect on the Clauss assay but may affect PT-Fg through their effect on the PT. A prolonged thrombin time with a normal reptilase time is the classic signature of heparin or a direct thrombin inhibitor, since reptilase is not inhibited by antithrombin, and this simple pairing resolves many otherwise puzzling results [7]. Activated charcoal-based removal products can be used to eliminate direct oral anticoagulant interference where the drug cannot be avoided.

Other interferences include high concentrations of fibrin degradation products, which inhibit polymerization and lower the measured activity; paraproteins; and the sample factors noted in Section 4.3. Very high fibrinogen concentrations may also fall outside the calibrated range and require re-assay at a different dilution.

### 4.7. Reference Intervals

Because the diagnosis of hypofibrinogenemia is defined relative to the lower reference limit, the reference interval is not a background detail but part of the diagnostic criterion. Reference intervals must be established or verified locally for each reagent, analyzer, and population, and quoted with their source; the use of a single interval for both Clauss and PT-derived methods, or the transfer of a published interval without verification, is not acceptable practice [7].

Physiological variation must also be accounted for. Neonatal fibrinogen concentrations are lower than adult values, and the neonatal molecule differs functionally, so that a diagnosis of mild hypofibrinogenemia should not be made on a neonatal sample alone. Fibrinogen rises progressively during pregnancy, commonly to 4–6 g/L at term, which can bring a woman with mild hypofibrinogenemia into the reference interval and conceal the diagnosis. Concentrations rise with age, with obesity, with smoking, and with any acute-phase stimulus.

For the avoidance of the inconsistency that has characterized some accounts of these disorders, this review uses laboratory-specific reference intervals throughout, with an adult interval of approximately 1.5–4.0 g/L quoted only illustratively and does not assert a universal lower limit.

### 4.8. Discordant Results: Interpretation and Escalation

Discordance among the laboratory results, or between the laboratory and the patient, is informative rather than inconvenient, and should trigger investigation rather than repeat testing until an acceptable number is obtained. Four patterns recur. An unmeasurable Clauss value with a detectable antigen suggests either severe hypofibrinogenemia at the analytical margin or a qualitative defect, and requires a sensitive antigen method and re-assay at reduced dilution. A normal PT-Fg with a low Clauss value suggests dysfibrinogenemia or PT-Fg overestimation. A low fibrinogen with a normal thrombin time is internally inconsistent and points to interference or pre-analytical error. Finally, a severe bleeding phenotype with only mildly reduced fibrinogen should prompt consideration of a coexisting hemostatic defect rather than attribution to the fibrinogen result alone.

Two worked examples illustrate how these patterns are resolved in practice.

Example 1: unmeasurable activity with a detectable antigen. A six-week-old infant is investigated after prolonged bleeding from the umbilical stump. The Clauss fibrinogen is reported as undetectable, the PT and aPTT are unclottable, and an immunoturbidimetric antigen assay returns 0.18 g/L. Two readings are possible: severe hypofibrinogenemia, with activity lying below the analytical limit of the local Clauss assay, or afibrinogenemia, in which the antigen figure is an artifact at the bottom of an assay not validated for that range. The two are separated analytically, not clinically. The Clauss assay should be repeated at a reduced plasma dilution, which extends the measuring range downward and will return a numerical value if any clottable fibrinogen is present. The antigen should then be re-measured by a method with a documented lower limit of quantification in this range, such as ELISA, rather than by a turbidimetric assay calibrated for normal plasma. If reduced-dilution activity remains unmeasurable while a sensitive antigen method confirms a low but real value, the diagnosis is severe hypofibrinogenemia rather than afibrinogenemia—a distinction that changes the expected inheritance pattern, the counseling given to the parents, and the priority of copy-number analysis. Note that the Fg:C/Fg:Ag ratio should not be calculated at all in this situation: dividing an unmeasurable numerator by a denominator sitting at the limit of quantification of a second assay yields a number without meaning.

Example 2: low Clauss activity with a normal prothrombin time-derived result. A 28-year-old woman is referred after a preoperative screen showed a Clauss fibrinogen of 0.7 g/L, while the same analyzer simultaneously reported a PT-derived fibrinogen of 2.4 g/L. The PT is 14.1 s, and the thrombin time is prolonged at 26 s. Here, the discordance is itself the finding rather than an error. A low Clauss value with a near-normal PT-derived value and a prolonged thrombin time is the characteristic signature of a qualitative defect. The reason is that the PT-derived method infers concentration from the magnitude of the optical signal change during clot formation, and therefore reports a structurally abnormal molecule as though it were functionally intact [52]. The correct next step is an antigen assay. An antigen of 2.3 g/L gives an Fg:C/Fg:Ag ratio of approximately 0.30, well below 0.7, placing the patient in dysfibrinogenemia and outside the scope of this review. Had the antigen instead been 0.9 g/L, the ratio of approximately 0.78 would have indicated hypofibrinogenemia, and the prolonged thrombin time would be explained by the low concentration alone. The generalizable point is that the PT-derived result, which is reported by default on many analyzers and is easily overlooked, carries real diagnostic information when it disagrees with the Clauss value—provided it is not mistaken for the fibrinogen concentration itself.

Referral to a specialized hemostasis laboratory should occur before the diagnosis is finalized in any of these situations, and routinely where afibrinogenemia is suspected, where genotyping is contemplated, or where the result will determine the management of pregnancy or major surgery. Specialized laboratories offer reduced-dilution Clauss assays, validated antigen methods, reptilase and related tests, viscoelastic and clot-structure studies, and access to molecular diagnostics with appropriate interpretation.

### 4.9. Molecular Diagnosis

Genetic confirmation establishes the diagnosis definitively, enables cascade screening and counseling, and contributes to the accumulating genotype–phenotype dataset. It does not, on its own, predict clinical course.

Sanger sequencing of the three fibrinogen genes was historically the standard approach and remains appropriate where a familial variant is known. Targeted next-generation sequencing has now largely replaced it, whether as a three-gene assay or as part of a curated bleeding-disorder panel; the ISTH SSC maintains a curated list of genes with established disease associations, and restricting analysis to curated genes reduces the burden of uninterpretable findings [59].

Copy-number and structural-variant analysis is not optional in this disorder. Short-read sequencing is poorly suited to detecting large deletions, and the most common single cause of afibrinogenemia is a large FGA deletion. An NGS strategy without an orthogonal method for detecting copy-number change—read-depth analysis, multiplex ligation-dependent probe amplification, or microarray—will misclassify a proportion of patients as heterozygous when they are in fact compound heterozygous for a point variant and deletion. Larger chromosomal deletions encompassing the whole cluster can render a patient hemizygous, so that a single heterozygous variant produces a phenotype disproportionate to its apparent zygosity [37].

Variant interpretation should follow ACMG/AMP criteria [60]. Three features specific to this locus deserve attention: the numbering conventions discussed in Section 2.4; the presence of relatively common FGG variants that are not straightforwardly causative [37]; and the value of segregation and phase analysis within the family, which is often decisive where in silico evidence is equivocal. Variants of uncertain significance are common and should be reviewed in a multidisciplinary molecular hemostasis meeting attended by clinical scientists and the treating clinicians, reported transparently as uncertain and re-interrogated periodically as evidence accrues. The deposition of variants in shared repositories is the mechanism by which this uncertainty is ultimately reduced. Informed consent, incidental findings, and the implications of testing for relatives should be addressed prospectively [61].

### 4.10. Proposed Diagnostic Algorithm

Figure 4 sets out a stepwise approach that incorporates the analytical considerations above. It differs from previously published algorithms in three respects. Pre-analytical verification and the exclusion of acquired causes are given a step of their own, rather than being assumed. Analytical caveats are displayed alongside the step to which they apply rather than relegated to the text. Finally, confirmation, escalation to a specialized laboratory, and the handling of uncertain molecular findings appear as explicit steps.

## 5. Management

**Figure 5:**
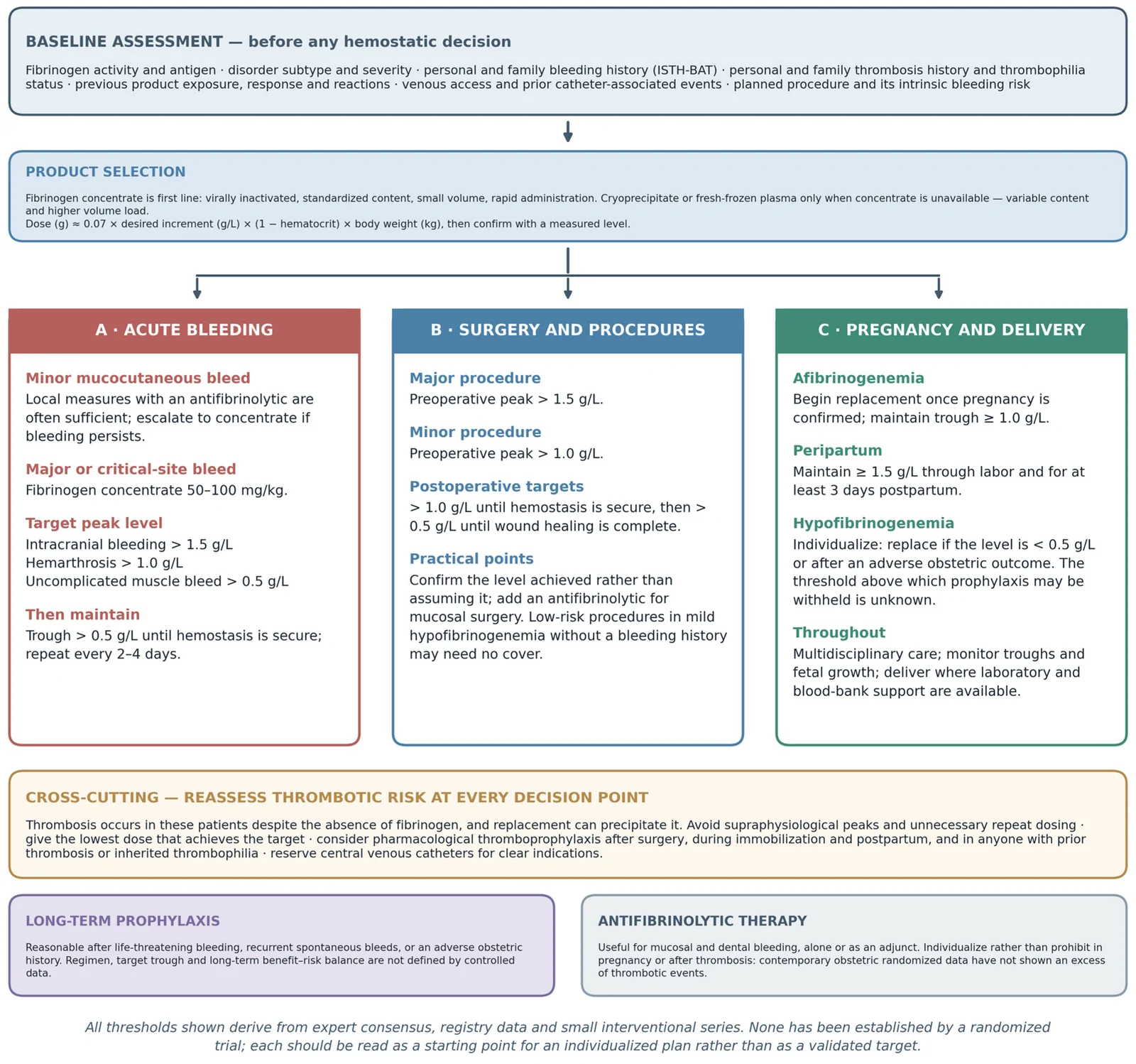
The management algorithm for quantitative fibrinogen disorders across acute bleeding, surgery, and pregnancy. The baseline assessment and product selection precede the three context-specific pathways. Thrombotic risk is shown as a cross-cutting consideration, reassessed at every decision point rather than as a terminal step. The thresholds shown are derived from expert consensus, registry data, and small interventional series, and should be treated as starting points for individualized care.

Management of the quantitative fibrinogen disorders rests on replacement of the missing protein, on the judicious use of antifibrinolytic agents, and on an unusually explicit balancing of hemorrhagic against thrombotic risk. Because randomized evidence is almost entirely absent, we state the basis of each recommendation as we go and summarize it in Table 3. Figure 5 presents the overall approach.

### 5.1. Replacement Products and Dosing

Fibrinogen concentrate is the treatment of choice wherever it is available. Compared with cryoprecipitate and fresh-frozen plasma, it offers a standardized and known fibrinogen content, multiple validated viral-inactivation steps, a small infusion volume, rapid reconstitution and administration, and a lower risk of allergic and transfusion-related reactions [62,63,70]. Efficacy and safety for on-demand treatment of bleeding and for surgical prophylaxis have been demonstrated in prospective studies in adults and in children [62,64,65], and long-term post-marketing analysis of one product has not shown an excess of thromboembolic events attributable to the concentrate itself. That analysis was generated by the manufacturer from its own pharmacovigilance database; however, its observational design and reliance on spontaneous reporting limit inference [66]. Cryoprecipitate and fresh-frozen plasma remain acceptable when a concentrate is unavailable, with the caveats of a variable fibrinogen content, larger volume load, and greater infectious and immunological risk [70,76]. Table 4 compares the licensed concentrates.

Dosing may be estimated from the conventional formula, dose (g) ≈ 0.07 × desired increment (g/L) × (1 − hematocrit) × body weight (kg), or from the product-specific formulae in Table 4. Both are approximations. Because in vivo recovery varies among patients and among products, the achieved level should be measured rather than assumed whenever the clinical stakes are high. Given a half-life of approximately four days, dosing every two to four days is generally sufficient to maintain a target trough [42,69].

### 5.2. Treatment of Acute Bleeding

Minor mucocutaneous bleeding in hypofibrinogenemia, and in patients with afibrinogenemia already receiving prophylaxis, can often be controlled with an antifibrinolytic agent and local measures alone, with escalation to a concentrate if bleeding persists [68]. Major or critical-site bleeding requires immediate replacement; an initial dose of 50–100 mg/kg of the fibrinogen concentrate is conventional [68,77].

The target levels are drawn from a European Delphi consensus: peak fibrinogen > 1.5 g/L for cerebral bleeding, >1.0 g/L for hemarthrosis, and >0.5 g/L for uncomplicated muscle bleeding without compartment syndrome, and, in other situations, a trough of >0.5 g/L maintained until hemostasis is secure [67]. These thresholds represent structured expert opinion, not trial evidence, and the tendency of some clinicians to aim substantially higher is not supported by data and may increase thrombotic risk [16,42].

### 5.3. Prophylaxis

Prophylaxis in this field is best described as an area of practice that has outrun its evidence. Secondary prophylaxis—regular replacement following a life-threatening bleed, recurrent spontaneous bleeding, or an adverse obstetric outcome—is widely practiced and is reasonable, and approximately one-third of patients in the QualyAfib cohort were receiving it [14]. Primary prophylaxis in patients who have not yet bled remains contentious: it has not been shown to improve outcomes, exposes patients to repeated venous access and to the thrombotic risk associated with replacement, and is not recommended in the absence of a bleeding phenotype [16,68].

The optimal regimen is genuinely unknown, and this should be acknowledged when prophylaxis is discussed with patients. Unresolved questions include the target trough, the dosing interval, the point at which prophylaxis should begin in a child with afibrinogenemia, and whether long-term prophylaxis alters the natural history rather than simply reducing bleed frequency. No randomized comparison of prophylactic regimens has been performed in this population.

### 5.4. Perioperative Management

Preoperative assessment should establish the disorder subtype and severity, the personal and family bleeding and thrombosis history, previous product exposure and response, and the intrinsic bleeding risk of the planned procedure, with hematology input obtained before rather than after the decision to operate.

For patients with afibrinogenemia, replacement is advised before any procedure regardless of bleeding history, targeting a peak > 1.5 g/L for major surgery and >1.0 g/L for minor procedures. Postoperatively, a level > 1.0 g/L should be maintained until hemostasis is secure and >0.5 g/L until wound healing is complete—the latter target reflecting the role of fibrin in tissue repair rather than in hemostasis [42,69]. Contemporary data support the adequacy of a peak of approximately 1.5 g/L before most major surgery [42], and prospective studies of concentrates for perioperative prophylaxis have reported satisfactory hemostatic efficacy across age groups [65].

Management should be individualized rather than applied uniformly, and two situations illustrate this. Patients with mild hypofibrinogenemia, no bleeding history, and a low-bleeding-risk procedure may not require replacement at all, and an antifibrinolytic with careful observation may suffice [69]. Conversely, a patient with moderate hypofibrinogenemia and a significant bleeding phenotype should be managed as though the deficiency were more severe than the number suggests. Where replacement is given, the achieved level should be confirmed rather than assumed. Secondary prophylaxis after surgery is reasonable where recurrent non-life-threatening bleeding has occurred [69].

### 5.5. Pregnancy and Delivery

Pregnancy in quantitative CFDs requires multidisciplinary management by a team combining hematology and maternal–fetal medicine expertise, ideally with a plan agreed before conception. ISTH SSC guidance now addresses this situation specifically [48].

For women with afibrinogenemia, replacement should begin as soon as pregnancy is confirmed, since first-trimester loss is the dominant risk and appears to be reduced by maintaining an adequate trough. A trough of ≥1.0 g/L throughout pregnancy, rising to ≥1.5 g/L peripartum, is the most widely applied target; the case-series data are consistent with better outcomes at these levels than at lower ones [48,71]. UKHCDO guidance recommends maintenance above 1.0 g/L throughout pregnancy, with escalation above 1.5 g/L peripartum [68]. Monitoring of trough levels at least monthly, together with ultrasonographic assessment of fetal growth and placental appearance, is appropriate.

Postpartum, the duration of replacement should be stratified by mode of delivery rather than applied as a single figure. The ISTH SSC communication distinguishes the two situations, suggesting that a fibrinogen level ≥ 1.5 g/L be maintained for three days after vaginal delivery and for five days after cesarean section [48]. The longer period after operative delivery reflects the surgical wound, the greater expected blood loss, and the later peak incidence of secondary postpartum hemorrhage.

Two qualifications belong with these figures. They are consensus suggestions rather than trial-derived thresholds, and the same source emphasizes individualization. More importantly, the puerperium is the period in which the competing risk is greatest: immobilization, the physiological hypercoagulability of the postpartum state, and supraphysiological fibrinogen peaks from replacement coincide, and postpartum thrombosis is documented in this population, including in women receiving prolonged replacement [71]. The duration should, therefore, be reviewed daily against actual blood loss, and replacement stopped when the bleeding has settled, rather than continued to a predetermined day count; thromboprophylaxis should be considered in parallel.

For women with hypofibrinogenemia, the position is less clear, and prophylaxis is genuinely controversial. Replacement is generally advised where the fibrinogen level is below 0.5 g/L or where there has been a previous adverse obstetric outcome. Above that level, and in the absence of adverse obstetric history, the threshold at which prophylaxis may safely be withheld has not been established, and close monitoring with a low threshold for intervention is the pragmatic approach [48].

The mode of delivery should be determined by obstetric indications. No evidence establishes the superiority of cesarean section over vaginal delivery in this population; the argument advanced for planned cesarean rests on avoiding prolonged labor and instrumental delivery in a fetus who may be affected [72] and is reasonable but not evidence-based. Where the fetus is known or suspected to be affected, invasive fetal monitoring and instrumental delivery should be avoided [48]. Postpartum hemorrhage remains common even with prophylaxis, so replacement and antifibrinolytic therapy should be immediately available. Postpartum thromboprophylaxis should be actively considered, weighing the thrombotic history, the intensity of replacement, and conventional obstetric risk factors [42,48].

### 5.6. Thrombosis and Thromboprophylaxis

Managing thrombosis in a patient with no fibrinogen is among the most difficult problems in hemostasis, and the honest position is that the practice is individualized because evidence is absent. Acute thrombosis generally requires anticoagulation alongside sufficient fibrinogen replacement to prevent hemorrhage, a combination that demands close monitoring and specialist input. In patients with mild or moderate hypofibrinogenemia, acute thrombosis is usually managed according to general population guidelines, modified by the bleeding phenotype [42].

For prevention, three measures are reasonable on mechanistic grounds and are widely applied. Supraphysiological peaks should be avoided and the lowest dose achieving the target used. Pharmacological thromboprophylaxis should be considered after surgery, during immobilization and in the postpartum period, and particularly in patients with previous thrombosis or coexisting thrombophilia. Central venous catheters should be reserved for clear indications, since catheter-related thrombosis is a recognized complication in this group [14,42].

### 5.7. Dental Procedures and Minor Surgery

No dedicated guidelines exist for dental treatment in quantitative CFDs, and the pragmatic approach has been to extrapolate from protocols established for hemophilia and von Willebrand disease [73]. Hematology consultation should precede oral surgery. A target fibrinogen level of at least 1.0 g/L is generally recommended before extraction, with the decision to give a concentrate before or after the procedure made case by case. Local measures—hemostatic dressings, sutures, or fibrin sealant—should be used, and patients should be given clear postoperative instructions to protect the clot. Drugs that impair hemostasis, particularly non-steroidal anti-inflammatory agents, should be avoided.

The role of tranexamic acid requires restatement, because the blanket caution that has appeared in the literature is no longer well supported. Tranexamic acid is effective for mucosal and dental bleeding and may be given orally, intravenously, or as a mouthwash. The recommendation that it be avoided in pregnancy and in patients with a history of thrombosis should be qualified. In the largest obstetric dataset available—an individual patient data meta-analysis of randomized trials including more than 54,000 women—tranexamic acid reduced life-threatening postpartum bleeding and provided no evidence of an increased risk of thrombosis [74], a finding consistent with the earlier WOMAN trial [75]. The appropriate position is, therefore, individualization rather than prohibition: tranexamic acid may reasonably be used during pregnancy and in the postpartum period in women with quantitative CFDs when the bleeding indication is clear. Caution remains warranted in patients with active thrombosis, recent thrombosis, or a strong thrombotic phenotype, and in the presence of hematuria from the upper urinary tract, where clot retention is a concern. This is a matter of weighing two risks in an individual, not of a categorical contraindication.

## 6. The Controversies, Uncertainties, and Limitations of the Evidence

We set out here the questions on which the field is not settled, since these are the points at which clinical practice most often diverges and at which further research would have the greatest value.

The prevalence discrepancy. Genomic analyses identify far more predicted-deleterious fibrinogen genotypes than clinical practice encounters [8,11]. Whether this represents a large population of undiagnosed mild deficiency, systematic overestimation of penetrance by in silico prediction, or both is unresolved [12,13]. Resolving it requires phenotyping of variant carriers identified in population biobanks rather than further database interrogation.

Genotype–phenotype prediction. Residual fibrinogen activity predicts bleeding at the group level but performs modestly in individuals, agreeing with bleeding severity in only about half of quantitative cases in registry data [31]. No validated predictive model exists. Progress requires prospective cohorts with standardized bleeding assessment, systematic genotyping, including modifier loci, and a sufficient sample size—achievable only through international collaboration.

Thrombosis: mechanism and causation. The mechanism of thrombosis in the absence of fibrinogen remains hypothetical, and whether replacement therapy causes thrombotic events or merely marks the patients most likely to have them cannot be determined from observational data in which treatment intensity tracks disease severity. A prospective registry capturing replacement exposure, peak levels, and thrombotic outcomes with adequate granularity would address this; a randomized comparison of replacement strategies would answer it definitively, but is unlikely to be feasible.

Prophylaxis. Neither the indication for primary prophylaxis, nor the optimal regimen or target trough for secondary prophylaxis, nor the age at which prophylaxis should begin in children with afibrinogenemia are established. This is the area where a randomized trial is most needed and most plausibly achievable, since a comparison of two prophylactic regimens does not require a placebo arm.

Pregnancy thresholds. The targets in Section 5.5 derive from consensus and small case series. The threshold below which prophylaxis is required in hypofibrinogenemia, and whether the peripartum target of 1.5 g/L is necessary or merely sufficient, are unknown. Prospective international registry data are the realistic route to an answer.

Antifibrinolytic safety. The thrombotic risk historically attributed to tranexamic acid in these patients was extrapolated rather than observed, and contemporary obstetric randomized data do not support it [74]. Whether that reassurance transfers fully to patients with a quantitative fibrinogen disorder and a thrombotic phenotype has not been tested directly.

Limitations of this review. This is a narrative review, and the search strategy underpinning it is set out in Section 1.3. We did not apply predefined inclusion criteria or assess the study quality formally, and selection of the literature reflects the authors’ judgment. The underlying evidence base is dominated by case reports, small series, and registries, and is subject to publication bias favoring severe and complicated cases—a bias likely to inflate reported rates of complications, particularly in the obstetric literature. The product information in Table 4 was current at the time of writing, but licensing differs between jurisdictions and changes. Finally, the four-layer framework proposed in Section 2.5 is a synthesis intended to organize existing observations and generate testable hypotheses; it has not been validated as a predictive model and should not be used as one.

## 7. Future Directions

Five priorities follow from the uncertainties above, ordered by tractability rather than ambition.

Prediction of phenotype. The central clinical problem is the inability to tell which patient will bleed catastrophically and which will thrombose. Progress requires deep phenotyping, using standardized bleeding assessment tools [50], comprehensive genotyping extending beyond the three fibrinogen genes to candidate modifier loci [41], and functional characterization of clot structure and thrombin generation, all applied within cohorts large enough to support modeling. The four-layer framework in Section 2.5 provides a structure for such an analysis.

Laboratory standardization. The analytical problems in Section 4 are soluble. Four priorities stand out. The first is international standardization of fibrinogen calibrators and harmonization between methods. The second is the extension of external quality assessment schemes to samples at very low fibrinogen concentrations, where current schemes perform least well. The third is the validation of protocols for measurement below the conventional limit of detection. The fourth is the evaluation of functional assays—clot structure, thrombin generation, and viscoelastic testing—as adjuncts that may correlate better with phenotype than concentration alone.

Personalized replacement. Pharmacokinetically guided dosing, using individual recovery and half-life rather than population formulae, is technically straightforward and has not been systematically applied. Defining the minimum effective trough for each clinical situation would reduce both cost and thrombotic exposure. A randomized comparison of prophylactic regimens is the single trial most likely to change practice.

Registries and collaborative infrastructure. No single center accumulates sufficient patients. Existing international infrastructure, including the Prospective Rare Bleeding Disorders Database [31], should be extended, with agreed core outcome sets covering bleeding, thrombosis, obstetric outcome, product exposure, and patient-reported outcomes, and linked to variant repositories so that the phenotype and genotype accumulate together. The systematic deposition of variants with phenotypic annotation is the mechanism by which the burden of uncertain variants will be reduced.

Emerging molecular therapies. Replacement therapy is effective but burdensome, and definitive correction remains distant. Fibrinogen is a considerably harder target for gene therapy than the single-chain coagulation factors: three separate genes must be expressed in coordinated stoichiometry, the product must assemble correctly within the hepatocyte ER, and the combined coding sequence exceeds the capacity of conventional adeno-associated viral vectors. Liver-directed approaches, messenger RNA delivery, and gene editing are all conceivable, and the recent clarification of the role of ERAD in fibrinogen biogenesis [28] is directly relevant, since any therapeutic strategy must satisfy the same quality-control machinery that governs endogenous assembly. Preclinical models of fibrinogen deficiency exist and are suitable for evaluating such approaches [4]. In the nearer term, recombinant fibrinogen and improved plasma-derived products with defined factor XIII content are more realistic objectives.

## 8. Conclusions

Congenital afibrinogenemia and hypofibrinogenemia are rare, clinically heterogeneous and, in their severe forms, life-limiting. They are best regarded as parts of a phenotypic spectrum arising from variants in the same three genes, while recognizing that they differ systematically in inheritance, in bleeding risk, and in management requirements.

Three conclusions warrant emphasis. First, diagnosis is a laboratory problem before it is a clinical one. The Clauss assay is the reference method but performs least well precisely where these patients lie; prothrombin time-derived fibrinogen is not an acceptable substitute for diagnosis, reference intervals must be local, and discordant results should be investigated rather than repeated. Molecular confirmation requires copy-number analysis and disciplined variant interpretation, and confirms the diagnosis without predicting its course.

Second, phenotypic variability is not noise. It reflects identifiable layers of modification—at the locus, within hepatocyte quality control, through trans-acting genetic modifiers and through acquired factors—several of which are now mechanistically accessible and one of which, the iatrogenic layer, is directly modifiable.

Third, the management recommendations in this field should be transmitted with their evidential status attached. Fibrinogen concentrate is the treatment of choice, and the target levels in Section 5 are reasonable, but almost all of them rest on expert consensus rather than trial data. Presenting them as established discourages the individualization that these heterogeneous patients require and obscures the questions that most need answering: how to predict the thrombotic phenotype, and how prophylaxis should be given. Answering either will require the international collaboration that the rarity of these disorders makes both difficult and indispensable.

## Figures and Tables

**Table 1:** Comparative features of congenital afibrinogenemia and congenital hypofibrinogenemia.

Feature	Afibrinogenemia	Hypofibrinogenemia
Genotype	Biallelic (homozygous or compound heterozygous) variants; ~98% null alleles	Monoallelic variant; predominantly missense
Inheritance	Autosomal recessive	Autosomal dominant (variable expressivity)
Commonest locus	FGA (~85% of cases)	FGB or FGG
Fibrinogen activity	Below the analytical limit of detection	Below the lower reference limit; severe < 0.5, moderate 0.5–0.9, mild 1.0 g/L to lower reference limit
Fibrinogen antigen	Undetectable	Reduced in proportion (Fg:C/Fg:Ag ≥ 0.7)
PT, aPTT, TT	Grossly prolonged, often unclottable	Normal or prolonged according to level
Usual age at presentation	Neonatal period (umbilical stump bleeding)	Any age; frequently incidental in adulthood
Spontaneous bleeding	Characteristic, may be life-threatening	Uncommon except in severe deficiency
Typical bleeding pattern	Mucocutaneous, musculoskeletal, intracranial, umbilical	Provoked and localized; menorrhagia prominent
Wound healing	Impaired	Usually normal
Reported thrombosis	~18% in a large international cohort	Reported but less frequent
Hepatic storage disease	Not described	Recognized subgroup, chiefly FGG variants
Bone cysts, splenic rupture	Reported	Not characteristically reported
Pregnancy loss	High; replacement usually required from confirmation of pregnancy	Variable; individualized approach
Routine prophylaxis	Considered after major bleeding or adverse obstetric history	Not routinely indicated
Perioperative cover	Required regardless of bleeding history	Guided by severity, procedure and bleeding history

Fg:C, fibrinogen activity; Fg:Ag, fibrinogen antigen; PT, prothrombin time; aPTT, activated partial thromboplastin time; TT, thrombin time. The severity thresholds follow the ISTH SSC classification [6]; cohort data from references [14,30,31]. The reference intervals are laboratory-specific and must be locally verified.

**Table 2:** Analytical pitfalls in fibrinogen measurement, with their effects and mitigation.

Source	Effect on the Measured Result	Mitigation
Underfilled citrate tube	Falsely low activity; prolonged clotting times	Redraw; verify fill volume
High hematocrit (>0.55)	Falsely low activity	Adjust citrate volume for hematocrit
Sample drawn from a heparinized line	Falsely low Clauss fibrinogen	Redraw peripherally; thrombin time with reptilase time
Unfractionated heparin	Falsely low Clauss value, reagent-dependent	Use a heparin-neutralized reagent; reptilase time
Direct thrombin inhibitors (dabigatran, argatroban, bivalirudin)	Falsely low Clauss value; prolonged thrombin time	Reptilase time normal; activated charcoal removal; antigen assay
Direct factor Xa inhibitors	Little effect on Clauss; PT-derived value affected	Use Clauss rather than PT-derived method
High fibrin degradation products	Inhibition of polymerization; falsely low activity	Interpret with D-dimer; exclude DIC; antigen assay
Lipemia, icterus, hemolysis	Interference with optical endpoint detection	Repeat on a mechanical-endpoint analyzer
Paraproteinemia	Unpredictable interference in either direction	Antigen assay; specialized laboratory review
Result at or below the limit of detection	Underestimation; “undetectable” is analytical, not biological	Re-assay at reduced dilution; sensitive antigen assay
Calibrator and reagent differences between laboratories	Results not interchangeable between centers	Monitor serially on one platform; local reference intervals
PT-derived fibrinogen used as the primary method	Systematic overestimation, greatest at low levels	Diagnose with the Clauss method and antigen assay
Reference interval transferred from another laboratory	Misclassification at the lower limit	Verify reference intervals locally and cite their source
Neonatal or pregnancy sample	Physiologically lower or higher values	Repeat outside the physiological state before classifying

PT, prothrombin time; DIC, disseminated intravascular coagulation. Compiled from references [7,51,52,53,58].

**Table 3:** The evidence underpinning the principal management recommendations.

Recommendation	Basis of Evidence	Strength	Key References
Fibrinogen concentrate as first-line replacement	Prospective single-arm trials, pharmacokinetic and post-marketing data	Moderate	[62,63,64,65,66]
Peak > 1.5 g/L for cerebral bleeding; >1.0 g/L for hemarthrosis; >0.5 g/L for uncomplicated muscle bleeding	Structured expert consensus (Delphi)	Low—consensus only	[67]
Trough > 0.5 g/L until hemostasis is secure	Expert consensus	Low—consensus only	[67,68]
Preoperative peak > 1.5 g/L (major) and >1.0 g/L (minor)	Expert opinion supported by prospective surgical prophylaxis series	Low to moderate	[42,65,69]
Postoperative > 1.0 g/L until hemostasis, >0.5 g/L until wound healing	Expert opinion; mechanistic rationale	Low—consensus only	[69]
Cryoprecipitate and plasma reserved for when concentrate is unavailable	Comparative product characteristics; safety reasoning	Moderate	[63,70]
Secondary prophylaxis after life-threatening or recurrent bleeding	Observational and registry data	Low	[14,16]
Primary prophylaxis not routine in the absence of a bleeding phenotype	Expert opinion; absence of demonstrated benefit	Low	[16,68]
Replacement from confirmation of pregnancy in afibrinogenemia, trough ≥ 1.0 g/L	ISTH SSC guidance; case series	Low to moderate	[48,71]
Peripartum ≥ 1.5 g/L; postpartum ≥ 1.5 g/L for 3 days after vaginal delivery and 5 days after cesarean section	ISTH SSC guidance; expert consensus	Low to moderate	[48,68]
Prophylaxis in hypofibrinogenemia if <0.5 g/L or prior adverse obstetric outcome	Expert consensus; threshold not established	Low—contested	[48]
Mode of delivery determined by obstetric indication	Absence of comparative evidence	Very low	[48,72]
Thromboprophylaxis after surgery, during immobilization and postpartum	Mechanistic rationale; observational data	Low	[14,42]
Antifibrinolytics for mucosal and dental bleeding	Extrapolation from other bleeding disorders; obstetric randomized data on safety	Moderate for safety, low for efficacy in CFDs	[73,74,75]
Individualized rather than categorical restriction of tranexamic acid in pregnancy	Individual patient data meta-analysis of randomized trials	Moderate	[74,75]

Strength is expressed descriptively rather than by a formal grading instrument, because the underlying evidence is insufficiently homogeneous to support formal grading. “Low—consensus only” indicates a recommendation derived from structured or unstructured expert opinion without supporting comparative data.

**Table 4:** Licensed human fibrinogen concentrates.

	RiaSTAP/Haemocomplettan P	Fibryga/Octafibrin	Clottafact/FibCLOT
Manufacturer	CSL Behring, Germany	Octapharma, Switzerland	LFB Biomédicaments, France
Reconstituted concentration	20 mg/mL	20 mg/mL	15 mg/mL
Factor XIII content	Not specified	≈200 IU FXIII per g fibrinogen	Not specified
Storage	2–8 °C, up to 60 months	2–25 °C, up to 36 months	Up to 3 years
Stability after reconstitution	Use within 8 h	Up to 24 h at 25 °C	Use immediately
Adult dose when baseline level known	[target (mg/dL) − measured (mg/dL)] ÷ 1.7 (mg/dL per mg/kg)	[target (mg/dL) − measured (mg/dL)] ÷ 1.8 (mg/dL per mg/kg)	[target (g/L) − baseline (g/L)] × body weight (kg) ÷ recovery; adult recovery 0.043 (g/kg)/(g/L)
Adult dose when baseline unknown	70 mg/kg	70 mg/kg	0.05 g/kg
Hepatic impairment	No adjustment	No adjustment	No adjustment
Renal impairment	No adjustment	No adjustment	No adjustment
Reported half-life (hours)	78.7 ± 18.1	75.9 ± 23.8	69.3
Maximum adult infusion rate	5 mL/min	5 mL/min	4 mL/min

Product availability and licensed indications differ between jurisdictions; two concentrates are licensed in the United States, and the specific portfolio varies across Europe. Prescribers should consult the current summary of product characteristics in their own country. The half-life values are drawn from the respective product information and pharmacokinetic studies, and are not directly comparable among the products because the study designs differ.

## Data Availability

No new data were created or analyzed in this study. Data sharing is not applicable to this article.

## Supplementary Materials

The following supporting information can be downloaded at: https://www.mdpi.com/article/10.3390/hematolrep18050072/s1, Figure S1. Suggested Algorithm for Evaluating Suspected Fibrinogen Disorders [30,49,78,79].

## Abbreviations

ACMG/AMP: American College of Medical Genetics and Genomics/Association for Molecular Pathology; aPTT, activated partial thromboplastin time; BAT, bleeding assessment tool; CFD, congenital fibrinogen disorder; CNV, copy-number variant; DIC, disseminated intravascular coagulation; DOAC, direct oral anticoagulant; DTI, direct thrombin inhibitor; ER, endoplasmic reticulum; ERAD, ER-associated degradation; Fg:Ag, fibrinogen antigen; Fg:C, fibrinogen activity (clottable); FDP, fibrin degradation product; FFP, fresh-frozen plasma; FSD, fibrinogen storage disease; ICSH, International Council for Standardization in Haematology; ISTH, International Society on Thrombosis and Haemostasis; LMWH, low-molecular-weight heparin; LoD, limit of detection; NGS, next-generation sequencing; PT, prothrombin time; PT-Fg, prothrombin time-derived fibrinogen; SSC, Scientific and Standardization Committee; TT, thrombin time; TXA, tranexamic acid; UKHCDO, United Kingdom Haemophilia Centre Doctors’ Organization; VUS, variant of uncertain significance.
