# Supplementary material for: Congenital Afibrinogenemia and Hypofibrinogenemia: Genetic Basis, Pathophysiology, Laboratory Diagnosis, and Management
Source: Hematol Rep. 2026 Sep 30;18(5):72. doi: 10.3390/hematolrep18050072 (PMC13703998; doi:10.3390/hematolrep18050072)
Supplement: Supplementary file 1 [file hematolrep-18-00072-s001.zip › hematolrep-4340853-supplementary.pdf]

Figure S1. Suggested Algorithm For Evaluating Suspected Fibrinogen Disorders

Clinical suspicion: bleeding (personal/family history), thrombosis, obstetric morbidity, or incidental low/abnormal fibrinogen on coagulation testing

↓

Confirm and exclude common confounders:
repeat sample if needed; review anticoagulants (heparin/DTIs/DOACs) and major acquired causes (DIC/consumption, liver disease, dilution/massive transfusion).

↓

Initial laboratory evaluation (preferred):
PT, aPTT, thrombin time (TT) (+/- reptilase time), fibrinogen activity (Clauss; Fg:C), and fibrinogen antigen (immunoassay; Fg:Ag). Calculate Fg:C/Fg:Ag ratio.

Afibrinogenemia	Hypofibrinogenemia	Hypodysfibrinogenemia	Dysfibrinogenemia
Pattern: Fg:C <0.1 g/L (10 mg/dL) or below assay LoD<br>AND Fg:Ag undetectable.<br>Next: specialist review; confirm with FGA/FGB/FGG genetic testing.	Pattern: Fg:C < LLN (often <1.5 g/L)<br>AND Fg:Ag < LLN with Fg:C/Fg:Ag >=0.7.<br>Severity (Fg:C): mild LLN-1.0; moderate 0.5-0.9; severe 0.1-0.5 g/L.	Pattern: Fg:C < LLN AND Fg:Ag < LLN with Fg:C/Fg:Ag <0.7.<br>Next: specialist review; confirm with genetics; consider assay variability.	Pattern: Fg:Ag within reference interval (often >=1.5 g/L)<br>BUT Fg:C < LLN with ratio <0.7.<br>Alt screen: PT-derived/Clauss >1.43.

Notes: Use laboratory-specific reference intervals (RI) and assay limits of detection (LoD). Adult RI for functional fibrinogen is commonly ~1.5-4.0 g/L (varies by method/reagent; higher in pregnancy). Common screening cutoffs: Fg:C/Fg:Ag <0.7 (some labs 0.55); PT-derived/Clauss >1.43 when antigen is unavailable. Selected sources: Casini et al. J Thromb Haemost 2018 (ISTH SSC; DOI:10.1111/jth.14216) [30]; Casini, Hamostaseologie 2020 (DOI:10.1055/a-1182-3510) [78]; Casini et al. Acta Medica Martiniana 2022 (DOI:10.2478/acm-2022-0013) [79]; Simurda et al. 2021 (PMCID:PMC8622093) [49].

Reference

30. Casini, A.; Blondon, M.; Tintillier, V.; Goodyer, M.; Sezgin, M.E.; Gunes, A.M.; Hanss, M.; de Moerloose, P.; Neerman-Arbez, M. Mutational epidemiology of congenital fibrinogen disorders. Thromb. Haemost. 2018, 118, 1867–1874.

49. Simurda, T.; Asselta, R.; Zolkova, J.; Brunclikova, M.; Dobrotova, M.; Kolkova, Z.; Loderer, D.; Skornova, I.; Hudecek, J.; Lasabova, Z.; et al. Congenital afibrinogenemia and hypofibrinogenemia: Laboratory and genetic testing in rare bleeding disorders with life-threatening clinical manifestations and challenging management. *Diagnostics* **2021**, *11*, 2140.

78. Casini A. From Routine to Research Laboratory: Strategies for the Diagnosis of Congenital Fibrinogen Disorders. Hamostaseologie. 2020 Nov;40(4):460-466. doi: 10.1055/a-1182-3510. Epub 2020 Jul 9. PMID: 32645726.

79. Casini A. Diagnosis and Classification of Hereditary Fibrinogen Disorders. Acta Medica Martiniana. 2022;22(3):115-121. doi:10.2478/acm-2022-0013.
